# Editorial: Sound, Music, and Movement in Parkinson’s Disease

**DOI:** 10.3389/fneur.2016.00216

**Published:** 2016-11-28

**Authors:** Marta M. N. Bieńkiewicz, Cathy Craig

**Affiliations:** ^1^CNRS, ISM, Aix Marseille University, Marseille, France; ^2^School of Psychology, Queen’s University of Belfast, Belfast, UK

**Keywords:** PD, exercise therapy, music therapy, dance therapy, cueing

**The Editorial on the Research Topic**

**Sound, Music, and Movement in Parkinson’s Disease**

As editors of this special edition on sound, music, and movement in Parkinson’s disease (PD), we are delighted with the final collection of papers that have been published in this research topic. We would like to express our sincere gratitude to all of the authors who accepted the invitation to participate: a list that includes not only full time researchers but also clinicians, movement therapists, and dance professionals. We would like to thank all the reviewers, who gave up their time to critique the submitted articles and also the substitute editors who stepped in whenever needed. We thank the participants for their significant efforts, without which, these research projects would not be possible. Finally, we thank the Frontiers editorial and technical staff for their guidance and patience.

Research published over the last few years has reinforced the idea that activity and vigorous exercise have an important role to play in ameliorating the disease progression and preventing secondary health problems in PD (1–3). However, for patients, every movement requires a lot of effort and can easily cause fatigue, a phenomenon that often discourages patients from actively participating in physical therapy. Over the last decades, various groups of researchers have looked at how cueing (i.e., providing an external sensory framework such as a beat) can help support and improve the initiation and timing of movement. Stemming from the seminal work of Martin (4), therapies using visually, acoustically, and somatosensorially enriched environments have been reported to improve motor function, posture, and well-being in patients with PD. This ability to pick up and use external sensory information to guide and time movement appears to remain intact in people with PD unlike the ability to initiate and control intrinsically driven actions that appear to be more adversely affected by the disease [see Ref. (5) for plausible physiological model].

As a final collection, this special issue allows us to disseminate state-of-the-art knowledge on the functional deterioration of motor control and present novel behavioral interventions that aim to alleviate symptoms in PD. In particular, we are interested in forms of movement therapy that are sustainable, focused on improving quality of life in the long term and feasible even where resources are scarce. Our parallel aim was to push the Frontiers of our understanding to see how sensory information can afford and shape movement facilitation in PD and how our knowledge can feed into the design of tailored rehabilitation programs. This is why experts from the fields of auditory stimulation, neuroimaging, motor control, and dance therapy were invited to engage in a dialog on the current and future management of PD, suggesting possible new routes for therapy while outlining the limitations of our current scientific understanding. The end result of this international effort is presented in this e-book.

In order to organize the articles, we have divided this collection into four sub-themes.

## Gait and Lower Limb Movement Therapy

This section is opened with a review article from Hackney et al., which provides a comprehensive introduction to the core theme of this research topic. The focus is on neural substrates used for internally and externally guided movement in healthy participants and PD patients. The compendium of articles presented in this review provides an overview of the possible functional basis for the efficacy of pace-based rehabilitation interventions and also identifies future directions that merit additional investigation. The next article by Ashoori et al. continues along this theme and takes an in-depth look at the subject of rhythmic auditory stimulation (RAS). The authors not only discuss the underlying mechanisms for its therapeutic power but also deliver a synopsis of the benefits stemming from RAS-based interventions and other technological innovations that enable the creation of online cues that are adapted to the needs of each patient. This article is supplemented by the work of Maculewicz et al. and presents a roundup of the technological solutions currently available that make use of instrumented footwear and that can also be used for RAS. We would like to redirect readers, who are interested in finding out more about the progress of health informatics in PD management, to the recently published article of Espay et al. (6).

In terms of original research, two studies offer a promising outcome. First, the work of Pau et al. looks in detail at the spatiotemporal and kinematic changes in gait patterns following a therapeutic program that encompasses RAS training. Second, Ridgel shares an update on the novel developments in the high-cadence cycling therapy and its prevalence over static cycling; another significant step forward following the previous seminal work of Ridgel et al. (7, 8). This study brings hope that effective therapeutic regimes, which exploit our knowledge of high-intensity training, will be available for PD patients in the near future. Finally, two opinion papers compliment this section – Peterson and Smulders provide insight into the attentional aspects of parkinsonian gait and its implications for the design of cueing interventions, while Rodger and Craig discuss how there is a need to go beyond the metronome and consider the wealth of additional information that music or action relevant sounds (9) can offer in terms of sensory cueing. More complex and rich auditory structures are postulated to grant a more flexible mapping between the timing of each step and the temporal structure afforded by the beats, melodies, and chord progressions (these points are further discussed by Schiavio and Altenmüller).

## Motor Control Research

Difficulties with the spatiotemporal control of movement are the central theme of this section. Cameron et al. report on the effects of dopaminergic medication on two timing tasks that are based on rhythm discrimination and alignment. The authors conclude that medication supplementation and disease progression affect the ability to discriminate complex non-beat structures, but do not affect rhythm alignment ability compared to healthy adults. In addition, the authors find specific increases in the sensitivity to beat signals with dopaminergic medication. A study by Bieńkiewicz and Craig delivers some preliminary evidence for a correlation between severity of PD and difficulties in synchronizing movement to a simple beat, despite a preserved ability to apply as efficient movement strategies as healthy adults. Synchronization difficulty was found to be independent of movement amplitude and/or cognitive load. The interplay between the temporal processing and motor signs in PD is further discussed in the Perspective paper written by Schwartze and Kotz. These authors shift the focus from the functionality of the basal ganglia to a more widely distributed neural connectivity that includes the role of the cerebellum and the supplementary motor area in moderating the symptoms of PD. It is argued that impairment in temporal processing will have implications for the design of therapeutic interventions aimed at improving global motor function.

The closing contribution is a Method article presented by Torres et al. This paper demonstrates that by empirically estimating the family of probability distributions inherently present in the data, rather than *a priori* assuming a theoretical one, it is possible to extract the noise-to-signal ratio inherent in the data. This information, often called “noise” and traditionally smoothed out by averaging across epochs of data, actually contains a rich source of information about the integrity of the nervous system and progression of a neurological condition. The authors propose a novel platform for individual behavioral data analysis referred to as “precision phenotyping” and demonstrate its translatory power for the future development of personalized medicine as well as being a tool for distinguishing neurological conditions with often similar behavioral manifestations.

## Music Therapy

The music-based rehabilitation section is opened by a Hypothesis and Theory contribution by Schiavio and Altenmüller, which discusses the intricacies of the interactions between human cognition and the world from an embodied perspective. The authors point out the circular relationship between the body, brain, and the surrounding environment and the need to incorporate this into a rehabilitative context. From this perspective, motor rehabilitation interventions can be seen as reestablishing the lost relationship between the agent and the system, and not simple input/output dependencies. Music provides more than a simple timekeeping aid, by affording a variety of mind–body responses from self-regulation to sensorimotor coupling. Therefore, music motor therapy is not only effective from the mobility point of view but also from a psychological, socio-affective, and well-being standpoint. This message is repeated in two short Opinion papers that provide an additional overview of the power of music and RAS-based programs designed to alleviate parkinsonian symptoms. Mainka reinforces the idea that music offers a superior approach to cueing movement in PD as its stimulatory power exceeds simple pacing through the esthetic qualities of the music that induces affective changes in the listener, which impact on general well-being. The structured auditory signals offered through music are easy to memorize and allow for a carry over effect from training after the session has finished. Moreover, Raglio points out that strong methodological criterion should be employed for future studies investigating not only motor improvements but also psychological outcomes of these types of interventions to allow for a direct comparison with other exercise regimes and RAS programs.

## Dance Therapy

Apart from gait training, music is a canvas for dance-based rehabilitation in PD. The opening piece in this sub-theme is an Opinion paper by Dreu et al., which provides an essential introduction with a short summary of the body of research in this area. Evidence for the multi-faceted benefits of partnered dance is listed and includes augmented mobility, improved balance, and general improvements in well-being as the primary outcomes. In addition, the authors discuss the importance of an enriched environment for successful therapy along with the somatosensory cues available from bodily contact with another person during dance.

A further three original articles provide examples and guidelines for designing a feasible dance program for PD patients, with measures of psychological outcome being included as well as improvements in mobility. Blandy et al. disseminated their work on a partnered tango intervention with a proven safety and psychological health enhancement record. Similarly, Koch et al. describe an original non-partnered tango program and report increased well-being and self-efficacy measures for those who participated, reinforcing the key outcomes mentioned by Schiavio and Altenmüller. Following an embodiment approach, Batson et al. present a methodological paper that focuses on training agency in PD and propose an active improvisation dance program. In this scenario, patients are encouraged to react freely to verbal cues, mirroring the unpredictability of daily interactions. Finally, the last paper is by Marchant, who writes from the perspective of a professional dancer and teacher and discusses the important points that need to be considered when designing therapeutic dance interventions. The author draws on his own experience and the many research projects he has worked on with vulnerable groups such as PD patients.

Our hope for this collection of papers is that we shine new light on PD rehabilitation and provide inspiration to anyone who may benefit, whether they are researchers, practitioners, therapists, or simply the wider public. Last but not least, we hope PD patients reading this will feel motivated to actively seek out programs that use sound, music, and movement so that they can lead more active and fulfilling lives.

## Author Contributions

Both the authors (MB and CC) contributed equally to the ideas conveyed in the Editorial. MB wrote the main draft, which CC edited in terms of content and language. Both the authors approved the final manuscript and agreed to take responsibility for content contained.

## Conflict of Interest Statement

The authors declare that the research was conducted in the absence of any commercial or financial relationships that could be construed as a potential conflict of interest.
